# Migratory strategies of juvenile northern fur seals (*Callorhinus ursinus*): bridging the gap between pups and adults

**DOI:** 10.1038/s41598-019-50230-z

**Published:** 2019-09-26

**Authors:** Tonya Zeppelin, Noel Pelland, Jeremy Sterling, Brian Brost, Sharon Melin, Devin Johnson, Mary-Anne Lea, Rolf Ream

**Affiliations:** 1 Marine Mammal Laboratory, Alaska Fisheries Science Center, National Marine Fisheries Service, 7600 Sand Point Way N.E., Seattle, WA 98115 USA; 2 Ecology and Biodiversity Centre, Institute for Marine and Antarctic Studies, College of Science and Engineering, University of Tasmania, 20 Castray Esplanade, Hobart, TAS 7000 Australia

**Keywords:** Animal migration, Behavioural ecology

## Abstract

In species exhibiting differential migration by sex and age, understanding what differences exist, and the adaptive reasons for these differences is critical for determining how demographic groups will respond to environmental variability and anthropogenic perturbations. We used satellite-telemetered movement and diving data to investigate differential migration and its ontogeny in a highly migratory North Pacific Ocean predator, the northern fur seal (*Callorhinus ursinus*; NFS), with a focus on understudied juvenile (1- to 2-year-old) animals. We instrumented 71 juvenile NFS in two years (2006–07 and 2007–08) at three major North American breeding sites and compared their migratory strategies with pups and adults. Although sexual dimorphism is strong in adult NFS, only weak differences in body mass between sexes were found in juveniles, which had similar body mass to pups (~3–4 months). However, unlike widely-dispersed pups, juvenile male and female NFS dispersed in different directions, and used different habitats characterized by distinct hydrography and prey assemblages during migration, similar to breeding adults. Juvenile diving behavior differed only modestly among habitats and between sexes, consistent with weak differences in body mass. Evidence of habitat sexual segregation by juvenile NFS contradicts previous hypotheses that physiological differences predominantly drive the ontogeny of differential migration.

## Introduction

Long-distance annual migration is a common phenomenon in both marine and terrestrial species based upon individual adaptation to fluctuating resources^1^. Migration allows individuals to optimize resources and avoid predation or environmental exposure, but it also may expose individuals to new risks and costs. Differential migration occurs when separate age classes and/or sexes of animals exhibit varying migratory strategies and habitat selection^1,2^. One consequence of differential migration is that demographic groups within a population may have different levels of exposure to intra-annual variations in thermal stress, prey availability, predation pressure, altered habitats due to climate change, or more direct human interaction that could affect survival or reproductive success. Therefore, knowledge of differential migration within a species is key to understanding demographic trends, identifying sex and age-class specific critical habitats and developing effective conservation and management strategies for migratory populations.

The northern fur seal is a wide-ranging, highly migratory predator inhabiting the North Pacific Ocean and Bering Sea. The US population of NFS is comprised of the California stock (San Miguel Island [SM] and the Farallon Islands in California) and the larger eastern Pacific stock (EPS; the Pribilof Islands [PRB]; including St. Paul [SP] and St. George [SG] islands and Bogoslof Island [BG] in Alaska). Animals congregate on breeding islands during the summer months; pupping and breeding occurs during late June to late July^3^, followed by approximately 4 months of maternal care^4,5^. All age classes of NFS depart from breeding colonies in fall and undertake extensive pelagic migrations of thousands of kilometers to winter foraging areas in the Bering Sea and North Pacific Ocean^6–10^. The age and sex composition of migrating NFS varies among different regions^7,9–12^. Adult male NFS from the EPS primarily remain in the Bering Sea and North Pacific Ocean, whereas adult females migrate to the Gulf of Alaska, California Current, and Transition Zone Chlorophyll Front ecosystems^6,7,13^. Although data are more limited for the SM population, evidence suggests that adult females shift their distribution northwards and offshore during migration but remain in the California Current^14^. Pups from the EPS migrate throughout the central North Pacific Ocean and Bering Sea, foraging in predominantly offshore pelagic waters^15^, whereas pups from SM mostly remain in the California Current during the migration^16^. Some pups return to the breeding islands after the first winter, but most are not observed at breeding islands until two years of age^3,9,17^. Pelagic harvest and at-sea research collections from the late 1940s through the 1970s documented a more northerly distribution of juvenile males compared to juvenile females and found low numbers of both pups and juveniles in nearshore coastal waters suggesting they remain farther offshore than older animals^9,11,12^.

The reasons for, and consequences of, differential migration in NFS are poorly understood, though adult traits such as sexual size dimorphism and reproductive demands are hypothesized to be a primary driver^7^, as in other marine and terrestrial species^18,19^. Adult NFS exhibit a high level of sexual size dimorphism; adult males weigh between 200–250 kg while females are typically less than 45 kg^5^. Size dimorphism is present in NFS pups, although growth rates are similar between sexes for the first five years^20,21^. Reproductive traits also differ by sex; age of first reproduction for females is 4–5 years old whereas for males it is 8–10 years old because males must attain a large size before they are capable of defending territories and entering the breeding population^3,22^.

At PRB, the primary breeding site for the EPS, the population has declined by approximately 70% since 1975 while smaller populations on BG (approximately 400 km farther south) and SM (California stock) have been stable or increasing since 1980^23,24^. The reason for the current decline on PRB is unknown. The rate of mortality from weaning to 2 years old is a parameter that exerts a strong influence on NFS population stability, and variability in this parameter has been hypothesized to influence population trends^25,26^. NFS survival from weaning to 2 years old is variable and often low, with estimates of cohort survival for males from the EPS ranging from roughly 15% to 50%^25,27,28^. Knowledge of what causes mortality during this time is limited, because pup (4- to 12-month-old) and juvenile (1- to 2-year-old) NFS are highly pelagic and spend very little time on the breeding islands before two years of age^3,9,17^. Thus, it is likely that the lower survival of these age groups is related to the extended migratory phase of their life history.

Even though juvenile survival is thought to be a critical component driving the disparate population trends, relatively little is known about the foraging ecology and movement patterns of this age class. Descriptions of juvenile migration have relied on lethal collections of the species, and have not included tracking studies of individual juveniles during the migration. Further study of migratory behavior of both sexes across a range of ages is needed to elucidate the biological and environmental mechanisms that drive differential migration, and to assess the impacts of these patterns on juvenile survival and the associated demographic trends at the different breeding colonies. The aim of this study was to fill the gap in knowledge of migratory behavior of juveniles at three colonies with different demographic trends. More specifically, we used satellite location and dive data to compare pre-migratory foraging behavior, migration departure timing and routes, time spent in ocean ecosystems, and diving behavior of males and females from these colonies over two years. Juvenile migratory patterns are then compared with tracking data of NFS pups and adults to examine the age- and sex-specific ontogeny of migration.

## Results

**Figure 1:**
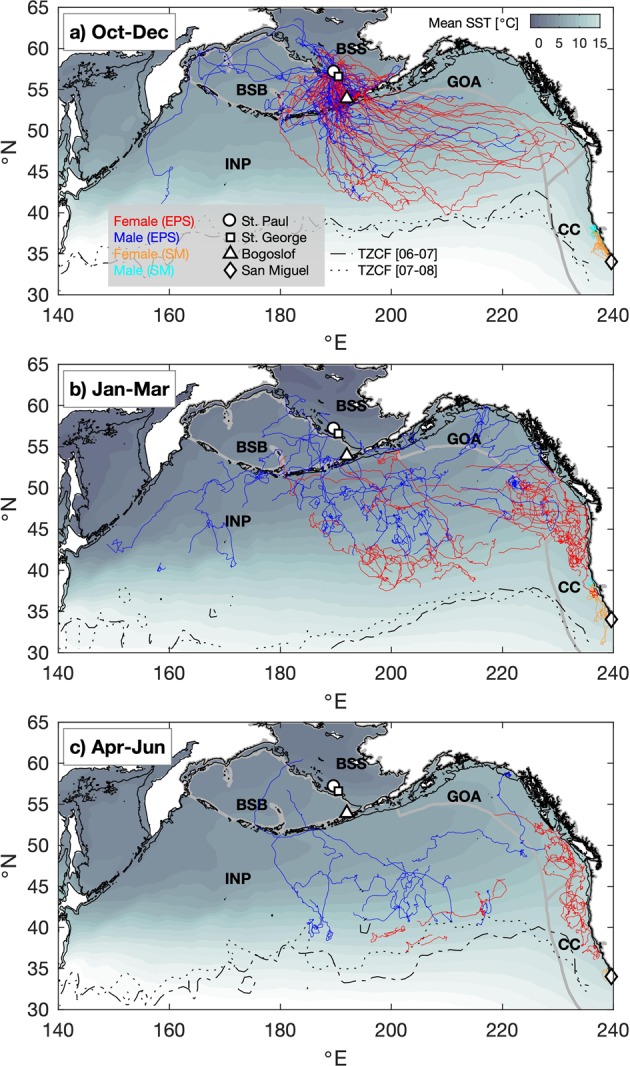
Composite seasonal migration of juvenile northern fur seals (NFS) from the eastern Pacific stock (EPS) and San Miguel Island (SM). In each season (**a–c**), migratory tracks from both years (2006–07 and 2007–08), colored by sex and stock, are overlaid on climatological seasonal average sea surface temperature (SST) from the NOAA Optimal Interpolation product (see Methods). Labels indicate major large marine ecosystems (Bering Sea Basin [BSB], Bering Sea Shelf [BSS], Gulf of Alaska [GOA], California Current [CC], and Interior North Pacific [INP]), boundaries of which are shown in light gray in each panel. Markers indicate tagging sites of juvenile NFS. Thin black lines in each panel denote the 200 m isobath (approximate continental shelf edge). Dotted and dot-dash lines indicate the positions of the Transition Zone Chlorophyll Front (TZCF) in each year, determined from seasonal averages of MODIS Aqua surface chlorophyll images.

We instrumented 71 juvenile NFS in 2006 and 2007 at three breeding islands: SP, BG, and SM (Fig. 1). Deployment durations for 68 satellite tags ranged from 27 d to 308 d (Table 1). Three remaining juveniles were excluded from the analysis because their instruments transmitted for less than 4 d. The body mass of juveniles ranged from 11.0 kg to 24.4 kg with a mean of 16.8 kg (Table 1). Juvenile body mass did not differ between males and females after controlling for the effect of island and year (linear regression, t-value = 0.89, *p* = 0.38). Juvenile data were compared with satellite tracking data from 124 adult females, 15 adult males, and 168 pups instrumented on PRB and BG in 12 separate years (Table 2), including 54 pups in 2006 and 18 adult females in 2006–07.

**Table 1:** Deployment year, site, and date, sample size (M/F: males/females), mean body mass (standard deviation, range), and mean satellite transmission duration (standard deviation, range) of juvenile northern fur seals with instruments transmitting >4 d.

Year	Deployment site	Deployment date	Juvenile M/F	Body mass male (kg)	Body mass female (kg)	Deployment duration (days)
2006	PRB (SP)	30 Sept–13 Oct	6/13	15.5 (1.2, 13.6–17.0)	15.4 (1.7, 13.2–19.6)	170.3 (67.6, 27.1–277.2)
	BG	4 Oct–7 Oct	5/5	16.8 (4.0, 12.2–21.8)	15.8 (3.7, 11.0–20.2)	188.2 (65.4, 26.8–244.7)
2007	PRB (SP)	5 Oct–15 Oct	16/17	17.5 (2.1, 14.6–23.2)	16.3 (1.5, 13.8–18.8)	169.9 (87.6, 30.6–308.1)
	SM	22 Sept	2/4	18.9 (0.1, 18.8–19.0)	22.5 (2.3, 19.2–24.4)	124.9 (66.0, 29.1–212.1)

Tags were deployed on St. Paul (SP) on the Pribilof Islands (PRB), Bogoslof Island (BG) and San Miguel Island (SM).

**Table 2:** Deployment year, site, and sample sizes (M/F: males/females) of pup and adult northern fur seals deployed on the Pribilof (PRB) and Bogoslof (BG) islands and the number of animals included in previous publications.

Year	Deployment site	Pup M/F	Adult M/F	Previously published pup M/F	Previously published adult M/F
1991	PRB		2/0		
1992	PRB		8/0		8^13^/0
1996	PRB	2/1		2^15^/1^15^	
1997	PRB	5/3		5^15^/3^15^	
2002	PRB		0/13		0/13^6,43^
2004	PRB		0/20		0/6^43^
2005	PRB BG	33/31 9/8	0/19 0/18	33^16^/31^16^ 9^16^/8^16^	0/2^43^ 0/7^43^
2006	PRB BG	22/19 9/4	0/7 0/6	22^16^/19^16^ 9^16^/4^16^	0/3^43^ 0/3^43^
2007	PRB		0/5		0/4^43^
2008	PRB		0/8		0/5^43^
2009	PRB		5/10		5^7^/10^7,43^
2015	PRB	0/17	0/18		

Juveniles from all sites made pre-migratory trips, in which individuals returned to land prior to embarking on their migration. Four instruments (3 EPS, 1 SM) collected only data on pre-migratory trips before ceasing transmissions. No complete migrations (a return to the colony after migratory dispersal) were recorded, however one animal instrumented in 2007 returned to within 63 km of its deployment rookery before its instrument stopped transmitting.

### Pre-migratory foraging trips

**Figure 2:**
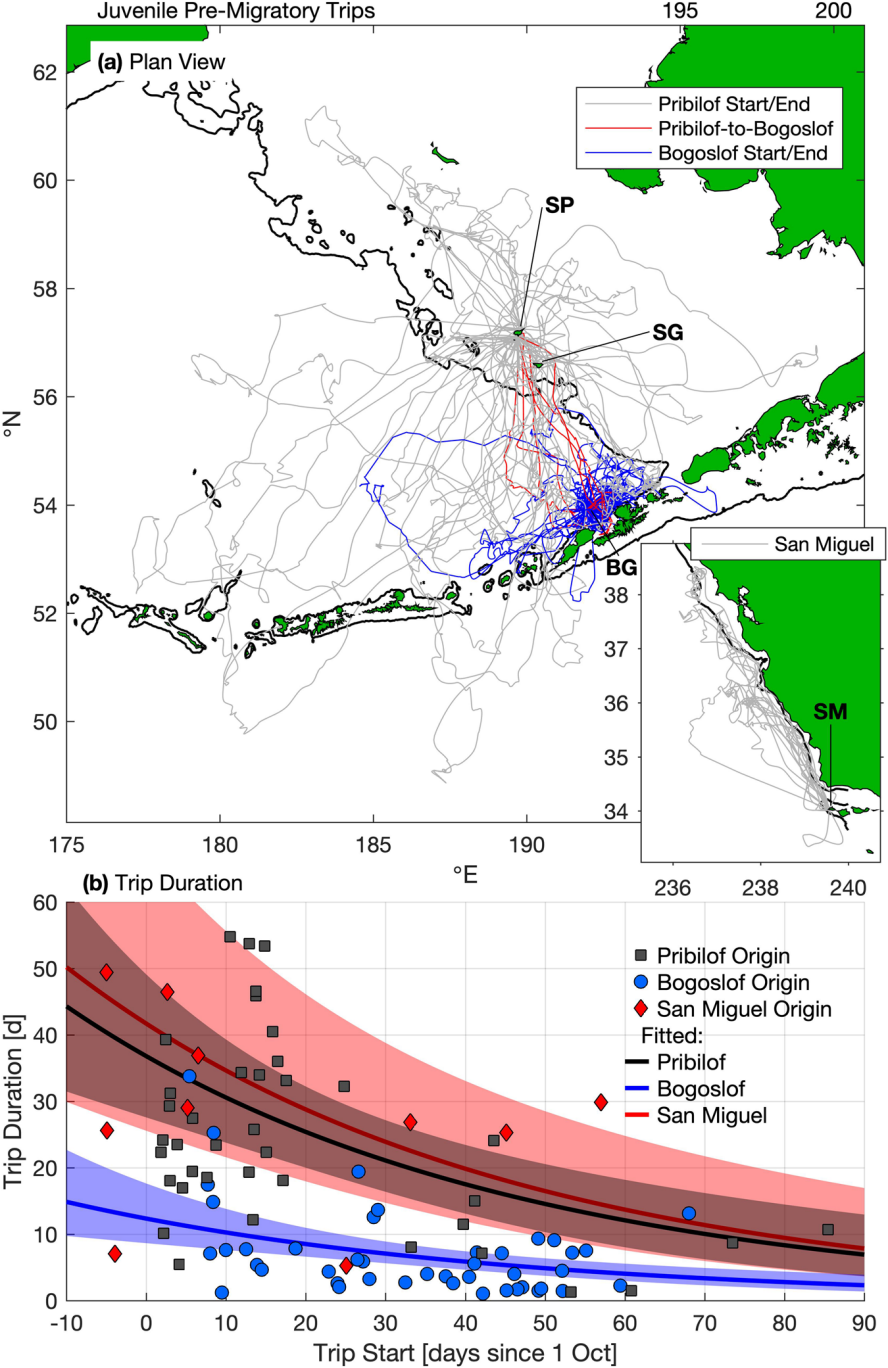
Pre-migratory foraging behavior of juvenile northern fur seals. Panel (a) shows pre-migratory trips, colored by start/end location for eastern Pacific stock (St. Paul [SP], St. George [SG], and Bogoslof [BG] islands) fur seals. Inset in panel (a) shows pre-migratory trips for San Miguel Island (SM) fur seals. Thin black lines indicate the 200 m isobath, as in Fig. 1. Panel (b) shows observed pre-migratory trip duration versus start day since 1 October, differentiated by site. Fitted values of mean trip duration versus site and day for juvenile females using a linear mixed-effects model (see Results section Pre-Migratory Foraging) are shown by solid lines; shading indicates 95% confidence interval on fitted values.

Juveniles made 106 pre-migratory foraging trips ranging from less than 1 d to 54.8 d; 92 trips of at least 1 d in duration were recorded (Supplementary Table S1). Trips from SM were mainly northward along and offshore from the continental shelf break (Fig. 2a). For EPS juveniles, trips ranged over the Bering Sea basin and shelf, and in some instances, south of the Aleutian Islands and into the Alaska Stream (Fig. 2a). Pre-migratory trips originating on BG were of shorter maximum distance and more spatially concentrated in the southeast Bering Sea basin than those originating on PRB (Fig. 2a, Supplementary Table S1). The longest pre-migratory trips for juveniles occurred early in the fall and originated from PRB or SM (Fig. 2b, Supplementary Table S1). Several animals with pre-migratory foraging trips originating on SP returned to an island other than their departure point (six to BG, three to SG, and one animal stopped at BG and SG), while BG and SM animals did not return to other islands prior to migrating.

Pre-migratory trips longer than 1 d were fit with generalized linear mixed-effect models (GLMM) with individual as a random effect (Supplementary Table S2); the top-ranking model indicated departure site (PRB, BG, SM) and day of departure since 1 October are important predictors of trip duration (Table 3). In the best model, foraging trips from BG were significantly shorter than those from PRB and SM and trip durations decreased 2% per day as departure date increased. The fitted values for mean trip durations were 8.4 d (95% CI: 6.5–10.8 d), 25.0 d (19.7–31.8 d), and 28.4 d (17.7–45.5 d) for trips originating from BG, PRB, and SM, respectively, on 21 October (the median observed pre-migratory trip departure date). Within pre-migratory trips, duration was significantly correlated with cumulative distance traveled (Fisher-Z test, *r*^2^ = 0.88; *p* < 0.0001), maximum distance from start point (Fisher-Z test, *r*^2^ = 0.74; *p* < 0.0001), and mean distance from start point (Fisher-Z test, *r*^2^ = 0.74; *p* < 0.0001).

**Table 3:** Top-ranking AICc selected generalized linear mixed-effects models for pre-migratory trip duration in days. Fixed effects considered included: sex, site at which the trip originated (Pribilof, Bogoslof [BG] or San Miguel [SM] islands), year, and days since 1 October on which each trip began (day).

Response variable	Covariate	Value	95% CI
Log (Trip duration)	Intercept	3.32	(3.03, 3.61)
	BG	−1.09	(−1.44, −0.74)
	SM	0.12	(−0.41, 0.65)
	Day	−0.02	(−0.03, −0.01)

Columns include estimated coefficients and 95% confidence intervals (CI) for each effect.

### Departure from land and the Bering Sea

Despite similar average departure dates among all EPS age classes, the range of juvenile dates was broader and contained later departures. The average departure date for EPS juvenile migration was 14 November in 2006 (standard deviation [SD] = 23.0 d; range: 3 October–23 December) and 1 November in 2007 (SD = 23.8 d; range: 4 October 2007–6 January 2008). Likewise, SM juveniles captured in 2007 had an average departure date of 22 November (SD = 39.4 d; range: 14 October–2 December). By comparison, average EPS departure dates were 13 November for pups (n = 163; SD = 8.2 d; range: 21 October– 11 December), 15 November for adult females (n = 124; SD = 8.2 d; range: 3 October–29 November), and 7 November for adult males (n = 15; SD = 14.1 d; range: 25 October–12 December).

**Figure 3:**
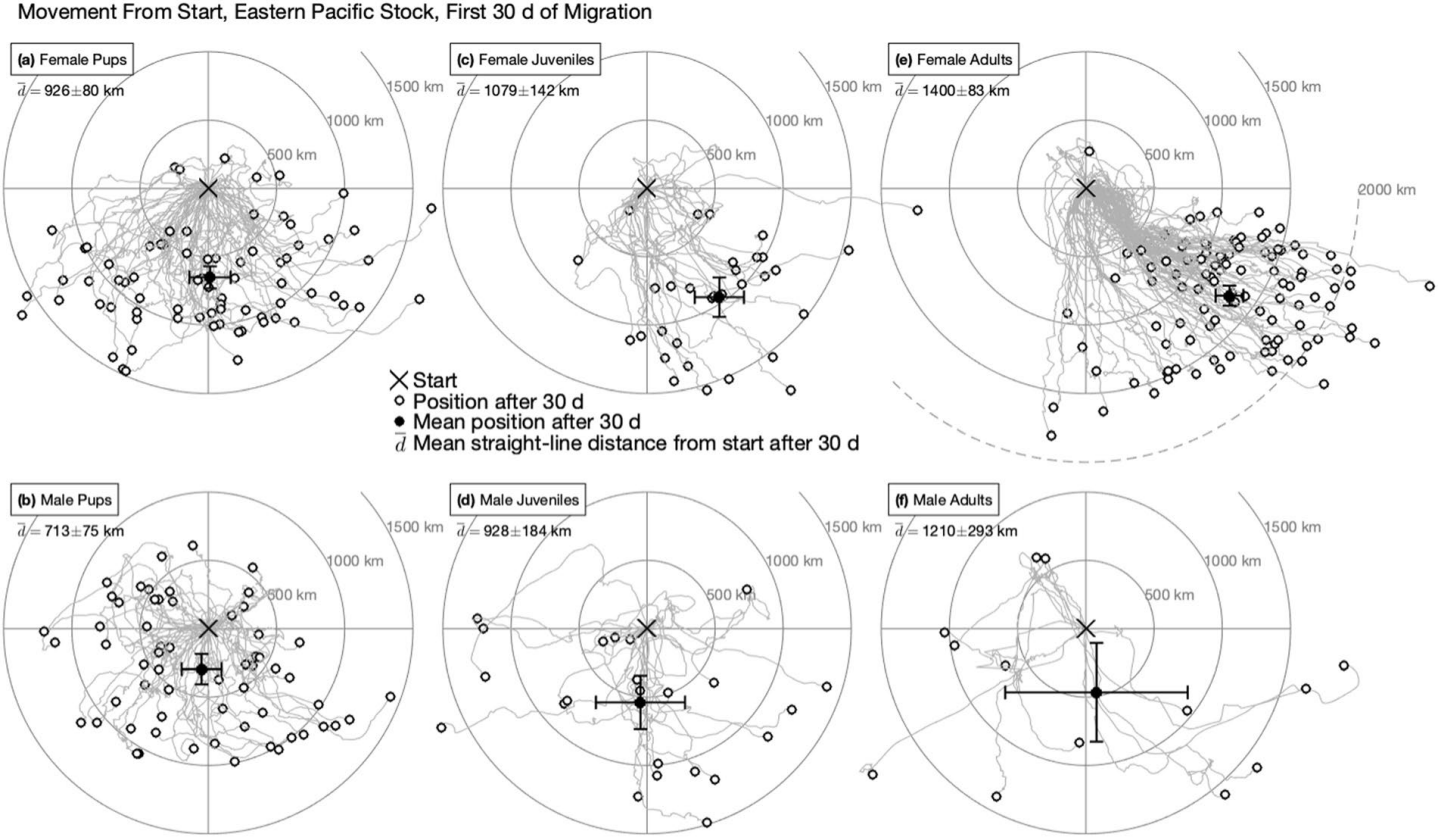
Displacement from start point in the first 30 d at sea for migratory northern fur seals of the eastern Pacific stock. Only tracks recording at least 30 d of migration are shown. Left column (**a,b**) shows pups, central column (**c,d**) juveniles, and right column (**e,f**) adults; top row (**a,c,e**) is females, whereas bottom row (**b,d,f**) is males. Within each group, the first at-sea point for each track is located at the origin (“x” marker), and displacements to the east (north) are along the positive x-(y-) axis. Filled white circles indicate the x/y position relative to the starting point for each animal at the end of 30 d; solid black circles indicate the average of these points for each group, with 95% confidence intervals (CI) indicated by whiskers. For each group, the average straight-line distance from the start point after 30 d and its CI is also shown.

To characterize movement and directional persistence during the initial dispersal phase, we calculated displacement and distance from departure site during the first 30 d of migration for EPS juveniles, pups, and adults (Fig. 3). Here displacement refers to the net east-west and north-south movement of each animal and distance is the total straight-line distance regardless of direction. After 30 d at sea, juvenile females were displaced an average 258 ± 238 km (95% CI) farther south and 580 ± 367 km farther east than males, despite traveling only 152 ± 227 km farther from their departure site. Female pups were 355 ± 136 km farther south than male pups after 30 d, but did not exhibit the same eastward displacement as female juveniles (juvenile females 518 ± 232 km farther east after 30 d) despite being a similar distance from their departure site (juvenile females 154 ± 161 km farther). The displacement of juvenile females to the south and east was similar to that of experienced adult females from the EPS. However, adult females were on average 320 ± 163 km farther from their departure point than juvenile females after the first 30 d at sea and were displaced 522 ± 205 km farther east. Male NFS did not exhibit changes in directional concentration from pup to juvenile, though juvenile males did travel a greater distance; male juveniles were on average 243 ± 221 km farther south and 215 ± 197 km farther from their departure site after 30 d than male pups. Within the limits of the data available for adult males, their mean distance from departure site, east displacement, and south displacement after 30 d at sea were not significantly different from juvenile males.

Following departure, all SM animals migrated to the north and remained within the California Current (Fig. 1). Fifty-seven of 59 EPS juveniles migrated south into the North Pacific Ocean (Fig. 1); tags on 2 male juveniles stopped transmitting while they were in the Bering Sea after only 2.25 and 14 d of migration. Twelve (3 females, 9 males) of the 57 EPS juveniles that entered the North Pacific Ocean returned to the Bering Sea at least once during migration. Generalized linear models for the rate of exit from the Bering Sea (i.e., a logistic model for the probability of entering the North Pacific Ocean in a 6-h interval, given that the animal is still within the Bering Sea in the previous interval) were fit to EPS juvenile tracks in 2006 and 2007 (Supplementary Table S3). The top-ranking model included site of departure (PRB vs. BG), sex, and multiple interactions between site, sex, and days at sea as predictors of the rate of exit (Table 4). For both sexes, departing from PRB was a predictor of a slower rate of exit from the Bering Sea, consistent with the greater travel distance from this site to the North Pacific Ocean in comparison to BG. Males from PRB departed at a slower rate than females, whereas differences among sexes were not evident at BG. In the fitted model, the mean time for the EPS juveniles to exit the Bering Sea was 15.2 d (95% CI: 12.0–18.2 d) for PRB females and 24.6 d (15.0–35.5 d) for PRB males. The corresponding values for BG were 8.8 d (4.4–17.9 d) for females and 7.1 d (4.0–9.0 d) for males.

**Table 4:** Top-ranking AICc selected generalized linear model for the rate of exit from the Bering Sea (p_exit_).

Response variable	Covariate	Value	95% CI
Logit (p_exit_)	Intercept	−3.54	(−4.24, −2.84)
	PRB	−1.76	(−2.83, −0.69)
	Male	−1.97	(−4.45, 0.51)
	PRB*Male	2.77	(0.09, 5.45)
	PRB*Days	0.10	(0.05, 0.15)
	Male*Days	0.38	(0.06, 0.70)
	PRB*Male*Days	−0.49	(−0.81, −0.17)

Effects considered included: sex, site of last departure (Pribilof [PRB] or Bogoslof [BG] islands), capture year, mass anomaly at capture (mass minus an average by sex), number of days at sea (days), and average north-south winds in the first 10 d at sea. Columns include estimated coefficients and 95% confidence intervals (CI) for each effect.

### Migratory habitat use

**Figure 4:**
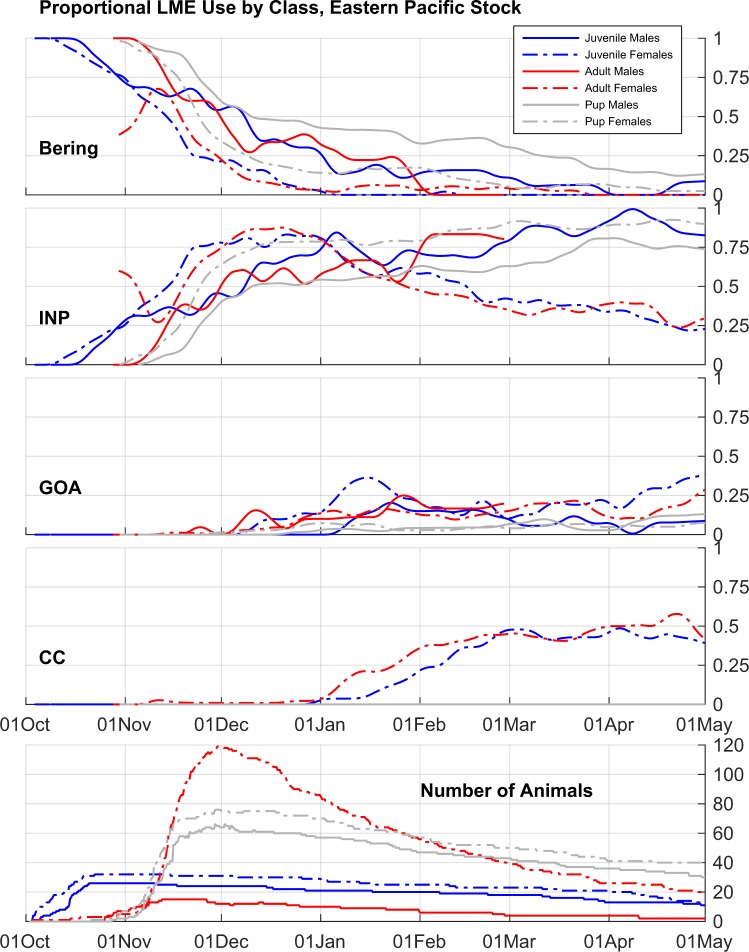
Composite proportional use of large marine ecosystems (LMEs) through time during the migration of eastern Pacific stock northern fur seals. Each of the first four panels corresponds to one LME or group of LMEs (Bering Sea Basin + Shelf [Bering], Interior North Pacific Ocean [INP], Gulf of Alaska [GOA], California Current [CC]); lines within these plots indicate the proportion of tagged animals in each age/sex class observed within that LME versus day of year. Lower panel indicates number of tagged animals in each class versus day of year. Observed proportions have been smoothed with a 5-d half-width triangular-weight running average filter for clarity.

We quantified juvenile habitat use using NOAA large marine ecosystems^29^ (LMEs; http://lme.edc.uri.edu/index.php/digital-data) which are delineated based on distinct hydrography, productivity, and prey assemblages^6^ (Fig. 1). Following Sterling *et al*.^7^, LMEs were grouped into aggregate LMEs representing the Interior North Pacific, Bering Sea Shelf, Bering Sea Basin, Gulf of Alaska, and California Current ecosystems (Fig. 1). Composite habitat utilization relative to LMEs versus day of year was calculated in order to illustrate the modal movement patterns of EPS juvenile NFS during the migratory period, and how this utilization may differ from adults and pups (Fig. 4). There was little difference in the wintertime distribution between years and deployment sites among juveniles from the EPS, but juveniles clearly exhibited differential habitat use by sex; juvenile females migrated to the Gulf of Alaska, California Current, and Interior North Pacific LMEs while juvenile males predominantly utilized the Interior North Pacific LME. By 1 March, 13 of 24 juvenile females from the EPS were in the California Current and two were in the Gulf of Alaska LME and they generally remained there until most tags stopped transmitting around 1 May. In contrast, on 1 March, none of the 18 EPS juvenile males were in the California Current LME and 2 were in the Gulf of Alaska LME. The remainder of EPS juvenile males were in the Interior North Pacific (n = 14) or Bering Sea LME (n = 2). Although sample sizes were small (2 females, 4 males), all SM juveniles remained in the California Current LME (Fig. 1).

The use of the California Current LME by EPS juvenile females closely paralleled that of adult females with a slight time lag that is consistent with the more rapid displacement of adult females from their departure point (Fig. 4). These observations are in contrast with those of female pups, which utilized the Interior North Pacific LME to a greater degree from 1 January onwards and did not use the California Current LME. EPS juvenile males appeared to utilize the Bering Sea LME to a lesser degree and the Interior North Pacific LME to a greater degree than male pups, with some limited use of the Gulf of Alaska LME in both age classes of males. The number of satellite-telemetered adult males was too small to draw firm conclusions regarding the juvenile-to-adult ontogeny in habitat use for male NFS, though the overall patterns appeared similar between these two age classes.

**Figure 5:**
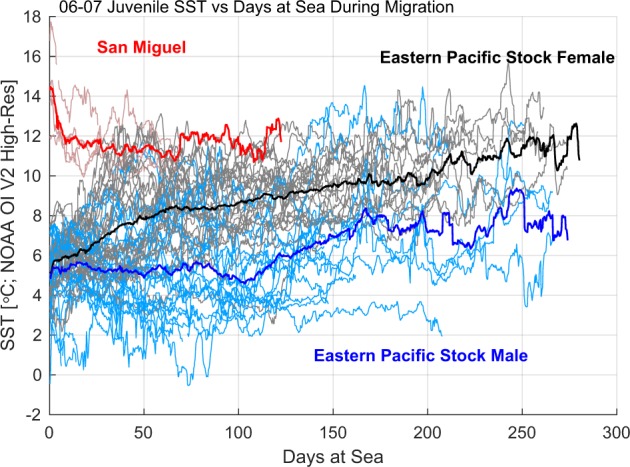
Sea surface temperature (SST) versus days at sea for male and female juvenile northern fur seals. Thin lines indicate individual tracks, which are colored by stock and, within the eastern Pacific stock, by sex. Solid lines indicate mean curves by group. SST along each track is determined by interpolating the NOAA Optimal Interpolation SST product to each individual’s estimated location versus time.

Juveniles of both sexes departing from SM experienced initial water temperatures of 12.1–17.5 °C, in contrast to −0.7–7.4 °C for individuals departing from PRB or BG (Fig. 5). Though there was variability within and between individual tracks, EPS females utilized warmer waters than EPS males, in particular following the first month at sea. Linear models were fit to the average sea surface temperature (SST) along each EPS individual’s track for tags recording the first 30 (T_30_) and 120 (T_120_) days of migration (Supplementary Table S4). The top-ranking model for T_30_ identified sex, year, and day of departure as predictors (Table 5). Animals departing in 2007 experienced colder conditions during the first 30 d, consistent with cooler SSTs observed on the mid-Bering Sea Shelf after mid-September in 2007 compared to 2006^30^. Juvenile males experienced average SSTs 0.39 ± 0.48 °C cooler than females over the first 30 d and there was a negative association between T_30_ and departure day (0.4 ± 0.1 °C decrease per 10-d delay) consistent with ocean cooling over time due to autumn and winter heat loss to the atmosphere^30,31^.

**Table 5:** Top-ranking AICc selected models for average SST in the first 30 (T_30_) and 120 (T_120_) days of juvenile northern fur seal migration.

Response variable	Covariate	Value	95% CI
Ave SST (T_30_)	Intercept	7.82	(7.27, 8.37)
	Male	−0.39	(−0.09, 0.09)
	Year (2007)	−0.62	(−1.10, −0.14)
	Day	−0.04	(−0.05, −0.03)
Ave SST (T_120_)	Intercept	8.09	(7.22, 8.96)
	Male	−2.29	(−3.18, −1.40)
	Day	−0.01	(−0.03, 0.01)

Fixed effects considered included: sex, site of last departure (Pribilof or Bogoslof islands), capture year (2006 or 2007), mass anomaly at capture (mass minus an average by sex), and day of departure since 1 October (day). Columns include estimated coefficients and 95% confidence intervals (CI) for each effect.

The best model for T_120_ included sex and day of departure as predictors of the SST experienced by juveniles (Table 5). Although mass, site and year were identified as predictors in other selected models, only sex was consistently identified in each of the selected models as a predictor of T_120_, indicating that it had strong explanatory power (Supplementary Table S4). Juvenile males experienced average temperatures more than 2 °C colder than females over the first 120 d. This is consistent with EPS male juveniles exiting the Bering Sea at a slower rate, and wintering at higher latitudes than females, where winter SST is colder^31^ (Fig. 1). As with T_30_, there was a negative association between departure day and T_120_ (0.1 ± 0.2 °C decrease per 10-d delay in departure; Table 5). The fact that the effect of departure date was greater in the model for T_30_ than T_120_ suggests that, as migrating animals dispersed, where each animal traveled was as important as the time of departure in determining the average ocean conditions they experienced over the first four months of migration.

### Migratory dive behavior

We collected histogram dive depth and duration data from 32 juveniles (16 females and 16 males) from PRB, BG, and SM during migration trips. A total of 1,615,586 dives greater than 2 m in depth and 805,317 dives greater than 15 s in duration were used in the analyses of migratory dive behavior. Of the total dives, 61% occurred within 6-h time periods classified as night (proportion daylight <0.2) and only 10% occurred during daytime (proportion daylight >0.8). A maximum dive depth of 175 m and maximum dive duration of 300 s were recorded during juvenile migration trips. However, most juvenile dives (87%) were less than 20 m in depth and 79% were less than 90 s in duration.

Linear models were used to examine migratory dive behavior. Dive depths of individual juvenile seals were best predicted by a model that included LME, season (days since October 1 scaled to 90-day units) and all two-way interactions among lunar fraction (illuminated area of the moon), LME and proportion daylight (Supplementary Table S5). A meta-analysis that pools inference across individuals further indicates that sex, stock and year were also important population level predictors of dive depth (Supplementary Table S6). As the percentage of lunar fraction increased, dive depth increased for both sexes in all LMEs (Supplementary Table S7). An increasing proportion of daylight was also a predictor of deeper dives in the Bering Sea Basin, Bering Sea Shelf and Interior North Pacific LMEs (Supplementary Table S7). Average estimated dive depths were relatively similar between sexes and ecosystems, however males dove slightly deeper than females in all ecosystems (Tables 6, S7). Although confidence intervals were wide due to individual variability, dives were deepest in the California Current and Gulf of Alaska LMEs and similar among all other LMEs (Tables 6, S7). Juveniles dove slightly deeper in 2007 than 2006 in all ecosystems and within the California Current LME dive depths of SM juveniles were shallower than EPS juveniles (Tables 6, S7).

**Table 6:** Predicted mean dive depths and durations (standard error) for juvenile males (M) and females (F) by large marine ecosystem (LME) groups for proportion of daylight = 0 (night), lunar fraction = 1 (full moon) and season = 1 November for the top model selected.

LME	Sex	Dive depth (m) (2006/2007)	Dive duration (s)	Night/day dives
Bering Sea Shelf	F	8.6 (1.0)/11.1 (1.2)	57.1 (4.0)	0.9/0.1
	M	10.4 (1.2)/13.4 (1.5)	70.0 (5.0)	0.7/0.1
Bering Sea Basin	F	11.5 (1.5)/14.8 (1.9)	72.9 (4.6)	0.9/0.1
	M	13.9 (1.8)/17.9 (2.3)	80.3 (4.7)	0.7/0.2
Interior North Pacific	F	10.9 (1.1)/14.1 (1.4)	66.3 (5.1)	0.7/0.1
	M	13.2 (1.3)/17.0 (1.8)	80.3 (6.2)	0.6/0.1
Gulf of Alaska	F	15.9 (2.7)/20.4 (3.5)	85.4 (9.2)	0.4/0.1
	M	19.2 (3.4)/24.7 (4.3)	86.5 (9.8)	0.5/0.1
California Current (EPS)	F	17.9 (2.1)/23.0 (2.7)	85.1 (7.4)	0.5/0.1
California Current (SM)	F	/12.8 (1.5)	71.3 (6.4)	0.3/0.1
	M	/15.5 (2.0)	108.5 (17.8)	0.1/0.01

Season is defined as days since 1 October scaled to 90-d units, night is defined as proportion of daylight < 0.2 and day is defined as proportion of daylight > 0.8. Proportions of day and night dives are calculated from depth records. All predicted values are for eastern Pacific stock (EPS) juveniles except for the California Current LME where predicted values are for EPS and and San Miguel (SM) juveniles. A meta-analysis included year as a predictor of population dive depth, therefore we provide predictions for both years, except for SM where data are only available for 2007.

The best model for dive duration of individual juvenile NFS included LME, lunar effect, daylight, season and an interaction between lunar effect and daylight (Supplementary Table S8). A meta-analysis further indicates that stock was an important population level predictor of dive duration and that the effect of LME varied by sex (Supplementary Table S9). Dive duration increased with an increase in the proportions of lunar fraction and daylight within each 6-h bin (Supplementary Table S10). As with dive depth, estimated dive duration of EPS females was longer than SM females (Table 6, Supplementary Table S10). Estimated dive durations were similar between sexes and ecosystems, but tended to be longer in the California Current and Gulf of Alaska LMEs (Tables 6, S10). Males dove longer than females in the Bering Sea Shelf and Interior North Pacific LMEs (Tables 6, S10). The interaction between lunar effect and daylight moderates the lunar effect during daytime for both dive depth and duration (Supplementary Tables S7 and S10). The relationship between season and dive depth and duration varied by individual; for some individuals there was a positive relationship and for some individuals there was a negative relationship. Consequently, the population coefficient for season was not significant (Supplementary Tables S7 and S10).

## Discussion

This study presents the first bio-logging data on the distribution and diving behavior of juvenile NFS during migration. By analyzing a robust sample size collected across multiple years and breeding islands, we provide a comprehensive examination of migratory behavior for this elusive age class. Understanding migratory strategies of juvenile NFS is an important missing link to understanding the driver of differential migration for NFS and critical to discerning the factors underlying the divergent population trajectories among the breeding islands.

Prior to migration, shorter juvenile foraging trips on BG compared with PRB were consistent with observed patterns of nursing adult females^23,32^, which suggests greater availability of near shore resources at BG compared to PRB in summer and fall. Juveniles from the EPS made longer pre-migratory trips than those previously recorded for pups^15^ and adult females^23^, often traveled out of the Bering Sea, and moved between rookery sites during pre-migratory trips. Juvenile foraging trips were likely longer than pups and adult females because pups do minimal independent foraging prior to their first winter migration^33^ and adult females must return to the rookery within a specific time frame to nurse their dependent pups. Older juvenile (3- to 6-year-old) male NFS also make longer-ranging foraging trips than parturient females and typically return to several different rookery sites during the season^34^. Likewise, other juvenile pinniped and seabird species have been observed to make longer duration foraging trips than adults^35–37^ and visit non-natal breeding colonies prior to migration^38,39^. Juveniles may be developing successful foraging strategies and learning the locations of profitable foraging grounds on these long ranging pre-migratory trips to utilize during both migration and future foraging trips^35,37,40^. Juveniles may stop off at non-natal breeding sites as a result of being young and naïve or this behavior may indicate the potential for some degree of dispersal. Juvenile NFS have been observed to haul out on various rookeries, but most eventually return to their native colonies to breed^9^, suggesting non-natal rookery visits likely represent exploratory behavior.

Mean migratory departure dates were similar among all age classes of EPS NFS, however juvenile departures were more variable and included later departures than pups and adults. Migratory departure by NFS from the Bering Sea in fall is thought to be linked to lower air temperature, storm frequency, wind speed and the southward progression of sea ice^7,11,16,41,42^. Dispersal may also be linked to the availability of accessible and reliable food resources during winter^6,15^. The greater variability in juvenile departure dates may reflect the relatively fewer constraints on their migratory behavior compared to adults that have reproductive costs to balance or newly weaned pups that are inexperienced and vulnerable to environmental pressures such as wind and storms^16^. Furthermore, migratory departure dates and sites are less definitive for juveniles than pups and adults due to their movement between rookeries prior to migration.

Despite differences in pre-migratory trip duration, once migration began, only weak differences in migratory patterns between juveniles from the two EPS sites (BG and PRB) with divergent population trends were evident. Juveniles from BG exited the Bering Sea more quickly than PRB juveniles, but this is likely an artifact of BG having a closer geographical proximity to the North Pacific Ocean. It is currently unknown if entering the North Pacific Ocean more rapidly is advantageous for juvenile survival. Otherwise, juvenile males and females from PRB and BG utilized similar ocean ecosystems. The long-ranging migrations of both EPS sites is in contrast to SM juveniles, which remained in the California Current LME for the observed portion of their migration. There was very little evidence for overlap between SM and EPS juveniles during migration, though SM tags stopped transmitting earlier than EPS tags. However, even after January, when juveniles from both SM and the EPS were in the California Current LME, EPS juveniles were distributed farther north than SM juveniles (Fig. 1).

EPS juveniles exhibited differential dispersal and habitat use patterns by sex similar to those observed in migrating adults^6,7,43^. In contrast, only minimal sexual segregation in dispersal and habitat use was observed during migration of EPS pups^15^. The degree to which adult males and females from SM segregate during their migration is unknown and the number of juveniles tagged on SM during this study is too small to evaluate differences in habitat use. Further evaluation of differential migration within SM NFS and their comparison to the EPS may offer important clues into the environmental and biological drivers of NFS migration.

Despite utilizing similar habitat as adults, juvenile diving was much shallower and of shorter duration than adults^7,44^ and only slightly deeper and longer in duration than those previously recorded for pups^8,15^. Studies of other otariids also found that dive depth and duration increase with age and 2-year-old juveniles do not yet exhibit the dive abilities of adults^45,46^. These results are consistent with expectations based on differences in body mass among age classes because oxygen storage increases with body mass enabling larger animals to dive longer and deeper^46,47^. The slight increase in dive depth and duration by males compared with females may also be related to differences in their physiological development. Even in NFS pups, males dove slightly deeper than females during periods of increased lunar fraction^8^. In California sea lions (*Zalophus californianus*), sex differences in oxygen stores were evident in young juveniles with no difference in mass^48^. For migrating NFS pups, dive depth did not change through time while dive duration decreased suggesting pups become more efficient divers through time^8^. We found this same pattern among some individual juveniles, but not the population as a whole.

Differences in dive depth and duration were relatively small between sexes and ecosystems for juvenile NFS; however, females dove shallower and, in some ecosystems, for shorter durations than males. Shorter dive durations may indicate juvenile females are mostly transiting through the Bering Sea and Interior North Pacific ecosystems. Like adults and pups, juveniles of both sexes dove mostly at night and dive depth and duration increased with an increase in the proportions of lunar fraction and daylight^7,8,43^. Common NFS prey such as myctophids and squid migrate from the deep scattering layer to the surface at night and the depths of these prey increase with light levels from both daylight and lunar fraction^49,50^.

As with adult females, dives by juvenile females were deepest, and daytime dives more frequent, in the California Current and Gulf of Alaska LMEs^7,43^. These ecosystems are characterized by high productivity^51,52^, a relatively shallow winter mixed layer^53^, fewer days with strong winds^7^ and consistent eddies and meanders^54,55^. The fact that SM juveniles utilize the California Current LME year-round suggests that prey concentrations in this habitat are reliable. More even diving of juvenile and adult females during both day and night suggests that these ocean conditions concentrate prey near the surface making them more accessible during the day, particularly in the California Current LME^7,43^. Deeper diving of adult females during day in these ecosystems is thought to be related to prey at the base of the mixed layer, which is shallower than in the Interior North Pacific LME^7,43^. Juveniles may not have the diving capability to consistently access this feature, but their diving pattern is trending towards what is observed in adults. The distribution of juvenile dives includes dives to the depth of the mixed layer, but the mean depths are shallower. The reason for the overall greater depths and duration of night dives in these ecosystems is unknown, but could be related to differing prey assemblages due to the upwelling characteristics of the California Current LME.

The Interior North Pacific LME is characterized by a deeper mixed layer and higher winds than the California Current and Gulf of Alaska LMEs. During daytime, adult male NFS dive just below the mixed layer in this region, suggesting increased prey concentrations at this depth^7^. The mixed layer depth is 100 m or deeper in many areas of the Interior North Pacific LME^53,56^, and likely exceeds the diving capacity of adult females and juveniles^7^. Although juveniles did most diving at night, males had a lesser proportion of night-time dives than females in this ecosystem, in a pattern again trending towards adult behavior.

Juvenile males in the Interior North Pacific LME tended to winter at more northern latitudes than juvenile females in the same LME (Fig. 1). It is unknown to what degree this segregation is present in adults. Juveniles wintering in the southern portion of this LME are able to access the Transition Zone Chlorophyll Front (TZCF; Fig. 1), an area of elevated productivity where westerly winds push nutrient-rich subarctic waters into the northern reaches of the subtropical gyre^57^. The TZCF is known to be an area of heavy use by adult female NFS^6^ as well as many other migratory marine top-predators^57–59^. Results here suggest juvenile males in the Interior North Pacific LME benefit from TZCF productivity mechanisms to a much lesser degree than females. Instead, they may gain certain advantages by wintering in closer proximity to the breeding colonies, which as adults may enable males to arrive at the breeding colony earlier to establish better territories and to time their arrival to be in the best possible condition to defend their territories^60^.

Male and female juveniles experienced significant changes in SST during their migration that were associated with the habitats they travelled through. For EPS females, SST increased throughout the deployment duration, reflecting a southward movement out of the Bering Sea into warmer waters of the California Current and Gulf of Alaska^31,61^. Some juvenile females from the EPS reached SSTs as warm as those of SM individuals after as little as one month at sea (Fig. 5). For juvenile males, within a wide range of individual variability, SST remained consistent for the first 3–4 months and increased thereafter. This reflects the males’ longer retention in the Bering Sea, delayed southward movement and concurrent seasonal cooling in these areas.

Metabolic costs for migrating juvenile NFS vary according to the water temperatures encountered in different regions and seasons. Lower critical water temperature, defined as the lowest possible temperature at which an animal does not have to expend additional energy for thermoregulation, is estimated to be between 4 °C and 10 °C for pups to 2-year-old NFS^62–64^. For older juvenile female NFS (age 2.75–3.5 years), no thermal costs were found at water temperatures ranging from 2 °C to 18 °C in winter, making them physiologically capable of utilizing much of the North Pacific Ocean and southern Bering Sea during migration^65^. EPS juveniles in our study, estimated to be 1 to 2 years old, experienced ocean temperatures between −0.7 °C and 7.4 °C suggesting that they expended additional energy for thermal regulation early in their migration. Energetic costs of thermal regulation are likely to be greater for EPS juvenile males, especially during the first 120 d of the migration when they are utilizing the Bering Sea and Interior North Pacific LMEs. By contrast, SM animals never experienced water temperature below 10 °C, thus affording an energetic benefit by remaining in the California Current LME. Little is known about differences in survival rates of juvenile NFS between sexes and among breeding sites. It has been hypothesized that EPS females have slightly higher survival rates from birth to age 3 than males^12,66,67^, but there is little direct evidence of this^26,68^; future research is necessary to determine the relationship between energetic demands and survival.

The similarities between EPS 1- to 2-year-olds and adults in the initial dispersal, rate of exit from the Bering Sea, and strong sexual segregation in winter habitat use, suggests a relatively rapid progression of migratory behavior from pup to adult in NFS. Differences in physiological and diving capabilities resulting from sexual size dimorphism of adult NFS, combined with differences in prey distribution among habitats, is thought to drive segregation of adult NFS during their winter migration^7^. According to this hypothesis, adult males remain in Interior North Pacific, Gulf of Alaska, or southern Bering Sea LMEs during the winter because they are physically capable of accessing aggregated deeper water prey and tolerating more severe environmental conditions than females^7^. Adult females are significantly smaller than males and have energetic requirements for gestation during the migration that may not be met in colder waters to the north^10^. The sexual segregation observed in juvenile NFS in this study was an unexpected result because size dimorphism and reproductive costs for juveniles are minimal. Contrary to adults’ strong sexual dimorphism, juvenile NFS mass did not differ at capture and was similar to pup body mass. Thus, if mass is a predictor of diving ability, swim speed, and thermal tolerance in NFS^69,70^, the juvenile data do not support the adult hypothesis^7^ that within the context of their life history, the interaction between physiological limitations and the environment acts to directly drive differential migration in NFS.

Several other hypotheses may explain the early differential migration and ontogeny of migratory patterns observed in NFS. One possibility is that differential migration allows juvenile NFS to meet energetic demands that differ between sexes before mass differences are observed. In other sexually dimorphic species, female and male pup and juvenile differences have been found in utilization and assimilation of resources before size dimorphism is strongly developed. For example, female Antarctic fur seal (*Arctocephalus gazella*) pups utilize milk differently than males resulting in proportionately higher body lipid reserves^71^. Differences in metabolism and energy expenditure have also been observed between male and female juvenile northern elephant seals (*Mirounga angustirostris*) before differences in body mass exist^72^. This disparity is thought to be related to the development of sex-specific metabolic strategies necessary for future breeding success^37,72^. Passive drift experiences of early life stages may also shape adult migration routes^73,74^. In sea turtles, male and female hatchlings that emerge at different times of the season may have different drift scenarios that lead to differences in adult migration routes^73^. NFS pups are influenced by the wind^16^, but it seems unlikely that juvenile and adult NFS migration routes are determined from these experiences since pups do not exhibit differences by sex in migratory departure date^16^ and do not have differential migration patterns by sex similar to those observed in juveniles and adults.

Juveniles may segregate in preparation for future reproductive roles^75^. Antarctic fur seal males and females foraged at different trophic levels and utilized different foraging locations at 1 to 2 years old, when size dimorphism and breeding constraints were minimal^76^. Juvenile female Antarctic fur seals foraged similarly to adults, while juvenile males transitioned to adult foraging patterns at a much slower rate^76^. Earlier sexual maturity and reproductive demands of females is thought to be linked to the foraging segregation observed in juvenile Antarctic fur seals^76^. In addition to migration, sexually immature male and female NFS exhibit divergent behaviors during the breeding season associated with their adult reproductive roles. Like adults, juvenile males arrive at the breeding sites earlier than females^77^, and juvenile males fast for long periods to socialize on land while females do not^78^. Juvenile female NFS display stronger fidelity to their natal site than juvenile males; earlier development of this homing behavior is thought to be associated with their earlier reproductive maturity^9^.

The resemblance of juvenile migration patterns to those of adults, despite their lack of sexual size dimorphism and reproductive demands, and their limited diving ability suggests there may be an innate or genetic component to their migratory behavior. Consistent with this, although pups from the EPS did not utilize the same winter habitats as juveniles and adults, they did exhibit differences by sex in early southward displacement, similar to juveniles. Experiments with starlings *(Sturnus vulgaris)* suggest that they are born with an innate compass heading to follow on their initial migration and their navigation skills develop with age, enabling older birds to compensate for displacement and migrate to specific geographic areas^79^. If NFS migration were an innate behavior, we would expect to see similar migratory patterns in all age and sex classes. Pups did not utilize the same winter habitats as juveniles and adults, but they may be more heavily influenced by environmental conditions such as wind and currents which are known to affect early animal migratory patterns in the open ocean^16,73,74^. Despite similar body mass of pups and juveniles when they were captured in October and November, pups may lose nearly one half of their body weight over the first winter^80^ further compounding their susceptibility to environmental conditions. As pups mature into juveniles and their swimming ability and navigational abilities improve, they have more control over the direction of their movements allowing them to travel to and stay in winter habitats utilized by adults.

It is important to note that all years were pooled when comparing migratory characteristics by age in this study, to increase the sample size and include adult males in our analysis. Since data were not collected consistently across years, interannual variability may affect these comparisons. However, examination of interannual patterns within the data, or limiting the comparison to the 2006–07 deployment years, suggests that the conclusions of this study are robust to this effect. A comparison of the eastward 30-d displacement by year in EPS females found some interannual variability, but an increase in eastward displacement with age was observed in all years when instruments were deployed on multiple age classes of NFS (Supplementary Fig. S1). Likewise, patterns of 30-d displacement (Supplementary Fig. S2) and LME use (Supplementary Fig. S3) in 2006–07 deployment years look similar to patterns with all years combined (Figs. 3 and 4). Though there are small differences in use of the California Current by adult females and in the Bering Sea by male pups in the more limited comparison, these do not impact the inferences of this study. Migratory patterns of adults and pups described here are also consistent with previous studies that used multiple data sources and observation methods, indicating these patterns are stable across years (e.g.^9–11,41,81^).

Understanding migratory strategies of juvenile NFS and how they affect their survival and future reproductive output is an important missing link to understanding what is driving the PRB population decline and essential for assessing how NFS populations might be influenced by, and respond to, changing conditions during migration. Juveniles may be more susceptible to changes than adults due to lack of experience, lower thermal tolerance, greater diving constraints (e.g. dive depth^82^), and the need to consume more food per unit body mass^83^. Although the exact reasons underlying differential migration by sex in NFS remain undetermined, they likely represent adaptations that maximize reproductive potential for individuals of each sex, involving risks and benefits of migration within a variety of North Pacific Ocean ecosystems. The consistency between these results and historical studies suggests a persistence of differential migration patterns through time across periods with varying climate, available resources, and human interaction. The impacts of anthropogenic climate change and human activities such as commercial fishing^84,85^ and entanglement in marine debris^86^ on juvenile migration warrant continued investigation, in particular given the evidence presented in this study that adult migratory patterns are observed from a young age. Juvenile migratory strategies identified in this work provide a framework for future quantitative study of environmental indices that relate to juvenile survival.

## Methods

All work was conducted in accordance with and under the authority of the United States Marine Mammal Protection Act (National Marine Fisheries Service, NMFS Permits 782–1708). At the time of this study there was not an additional requirement for review of procedures by an ethics committee. In 2010, a NMFS Institutional Animal Care and Use Committee was established for the Alaska Fisheries and Northwest Fisheries science centers and all experimental protocols utilized in this study were reviewed and approved by this committee.

### Tag deployment and animal locations

We deployed satellite tags in September and October of 2006 and 2007 on 31 male and 40 female juvenile NFS from the 3 breeding sites spanning the North American range: SP on PRB, BG and SM (Supplementary Table S11). Juveniles were captured using a hoop-net, sexed, weighed, and Kiwisat202 (Sirtrak, New Zealand), SPLASH or SPOT (Wildlife Computers, USA) satellite transmitters were attached to the dorsal pelage in the mid-dorsal region between the scapulae using a two-part quick set epoxy (Devcon, Riviera Beach, FL). Juveniles were estimated to be 1 or 2 years old based on weight, tooth eruption, and behavior^77,87^ (Table 1).

Kiwisat and SPLASH tags were duty cycled to transmit at-sea locations 6 h/d (01:00–04:00, 13:00–16:00), whereas SPOT tags were programmed to transmit locations all day (360 transmissions/d). In addition to location information, SPLASH tags also recorded dive data every second, which were summarized over 6-h periods (GMT 05:00–10:59, 11:00–16:59, 17:00–22:59, 23:00–04:59). Maximum dive depth and dive duration were assigned to one of 14 bins (depth (m): 2, 5, 10, 20, 35, 50, 75, 100, 125, 150, 175, 200, 225, 225+; duration (s):15, 30, 60, 90, 120, 150, 180, 210, 240, 270, 300, 330, 360, 360+).

Animal spatial locations were calculated by Service Argos Inc. Data were extracted and processed using WC-DAP software (V. 3.0.447, Wildlife computers). Duplicate records and all low-quality (class Z) locations were removed from the data. Location data were filtered using an algorithm based on swimming speed, distance between successive locations, and turning angles^88^ (ArgosFilter; swim speed = 3 m/s).

Start and end times for pre-migration foraging trips and migration were determined using both location and dive data. Pre-migratory trips were defined as trips in which animals departed from and returned to a rookery site. Migration departure was defined as the last location after which an animal did not return to land. Departure and arrival times of foraging trips and migration were back calculated from the first and last at-sea locations using a swim speed of 3 m/s.

Tracks were reconstructed by modeling the filtered location data using a continuous-time correlated random walk model^89^. Locations were interpolated at 1- and 6-h intervals. In order to define ecosystems utilized by juvenile seals during their migration, interpolated locations were spatially joined with the NOAA polygon cover LMEs of the World^29^ (http://www.lme.noaa.gov).

### Additional northern fur seal data

To compare juvenile migratory departure timing, initial dispersal, and LME use with that of pup and adult NFS, we compiled satellite location data from 124 adult females, 15 adult males and 163 pups collected from instrument deployments on SP, SG and BG in 12 separate years (Table 2). Capture methods, instrumentation, and data processing for these deployments are described in Ream *et al*.^6^, Sterling *et al*.^7^, and Pelland *et al*.^43^ for adult females, Loughlin *et al*.^13^ and Sterling *et al*.^7^ for adult males, and Baker^15^ and Lea *et al*.^16^ for pups. A subset of the results presented in this study for adults are previously unpublished data (Table 2) and methods for these deployments were consistent with those described for previously published data. Although there were subtle differences in instrument programming among age classes and years, all instruments were programmed to transmit location data at multiple time periods per day. In all cases, raw location data were filtered for outliers and fit with a switching state-space^90^ or continuous-time correlated random walk^89^ model that interpolate locations to at least 6-h intervals prior to use in analyses for dispersal characteristics and LME use versus time. While the choice of movement model, tag programming or Argos processing method could influence the details of predictions at hourly or daily scales, these are likely to be negligible for the time and space scales on which comparisons are performed in this paper.

### Pre-migratory foraging

Linear mixed-effects models were used to assess the relationship between pre-migratory trip duration with respect to sex, site at which the trip originated (PRB, BG, or SM), year, day since 1 October on which each trip began and interactions between sex, site, and start day. The response variable, trip duration, was log-transformed and only trips >1 d (n = 92) were included in the analysis. Models were built using the fitglme function in MATLAB (The Mathworks, Inc.). For all models, individual was included as a random effect.

### Departure from the Bering Sea

A censored known-fate survival (KFS) model was used to assess the rate at which EPS juveniles depart from the Bering Sea after leaving land following Lea *et al*.^16^. This model seeks predictors that increase or decrease the probability of a juvenile departing the Bering Sea during a 6-h interval, given that it has remained in the Bering Sea until that interval. The response variable for each individual within each interval was 1 for departure and 0 for no departure; individuals who have departed the Bering Sea or whose tags have stopped transmitting are removed. Candidate models were constructed using linear and interaction terms composed of the following predictors: sex, site of last departure from land (PRB or BG), capture year, mass anomaly at capture (mass minus an average by sex), number of days at sea, and average north-south winds in the first 10 d at sea. The latter predictor was constructed from estimates of surface (10 m height) winds interpolated along juvenile NFS tracks from the National Centers for Environmental Prediction/National Center for Atmospheric Research Reanalysis 1 (R1) product (https://www.esrl.noaa.gov/psd/data/gridded/data.ncep.reanalysis.surfaceflux.html), available at 6-h intervals on an approximately 2° × 2° global grid^91^. The KFS models were fit as generalized linear models (GLMs) with a binomial distribution and logit link using the fitglm function in MATLAB.

### Sea surface temperature

Sea surface temperature along juvenile tracks was estimated by interpolating SST values from the NOAA Optimal Interpolation V2 High-Resolution (OISST) dataset (https://www.esrl.noaa.gov/psd/data/gridded/data.noaa.oisst.v2.highres.html) to modeled locations at 6-h intervals. The OISST product is a blended estimate based on satellite and *in situ* data, produced globally on a 1/4° grid at daily temporal resolution^92^. For juveniles from the EPS, the average SST during the first 30-d (n = 55) or 120-d (n = 39) period was estimated. Linear regression models were constructed for these response variables with different combinations of linear and interaction terms composed from the following predictor variables: sex, departure site, year, mass, and day of departure past 1 October.

### Dive data analysis

Dive data were extracted using Wildlife Computers WC-DAP software (V.3.0.447). Dive data were plotted by individual and month to check for spurious observations and subsequently three 6-h dive histogram bins out of 15,036 dive depth and 15,052 dive duration histogram bins were removed. Dive records ≤2 m in depth and ≤15 s in duration were also removed because these bins were almost always full and likely represent travel rather than foraging dives. Maximum and mean dive depth (m) and duration (s) per 6-h period were calculated following Lea *et al*.^8^.

Linear models were used to examine the effect of lunar illumination fraction, proportion daylight, ecosystem (LME), and season on migratory dive behavior (mean dive depth and duration in each 6-h period). A mixed-effect model with random intercepts and slopes could, in principle, accommodate variation between individual seals; however, this approach proved computationally expensive due to large sample sizes and autocorrelated error structures (e.g., AR(1)). In lieu of this technique, we first fit models to each seal separately, implicitly allowing all intercepts and slopes to vary by individual. Individual-specific coefficients were subsequently combined using a random-effect multivariate meta-analysis to obtain population-level effects^93,94^. Individual-level characteristics were further incorporated into the meta-analysis to explore the effect of sex, stock, and capture year on diving behavior. Linear models were fit using generalized least squares in R package ‘nlme’^95^ and included an AR(1) correlation structure to accommodate temporal dependence in dive behavior. The ‘mvmeta’ R package was used to perform the multivariate meta-analysis and synthesize individual-level inferences^93^.

Proportion daylight (proportion of 6-h time bin within daylight hours) and lunar illumination fraction (illuminated area of the moon’s disk divided by the total area of the disk, 0–1 where the full moon is 1) within each 6-h time period were calculated following Sterling *et al*.^7^ and Lea *et al*.^8^. Season was calculated as days from 1 October scaled to 90-d units. Marine ecosystems were defined by grouping LMEs following Sterling *et al*.^7^ (see Results section Migratory Habitat Use).

### Model selection and validation

Statistical models for each response variable were ranked according to the Akaike Information Criterion (AIC) or by Akaike Information Criterion corrected for small sample size (AICc^96^). We considered models within 2 AIC units (threshold adjusted for sample size in the case of AICc) of the AIC-best model competitive, unless the competing model only differed by an additional (uninformative) parameter^97,98^. We evaluated all models using standard model checking procedures, including plots of residuals versus fitted values, fitted versus observed values, quantile-quantile plots, and autocorrelation functions.

## Supplementary information

Migratory strategies of juvenile northern fur seals (Callorhinus ursinus): bridging the gap between pups and adults

## Data Availability

The juvenile, pup and adult telemetry datasets generated and/or analyzed during the current study are available from the corresponding author on reasonable request. Telemetry data from adult females deployed in 2002 and 2009 can be accessed on the Integrated Ocean Observing System’s Animal Telemetry Network website: https://portal.atn.ioos.us/#metadata/254997/species. Telemetry data from adult females deployed in 2004 and 2005 are available at http://projects.nprb.org/#module-search?page=1&tagId=&q=0514&tags=&types=module%2Csensor_station%2Cproject. Environmental data used in this study (R1 winds, NOAA OI SST, proportion daylight, lunar fraction) and NOAA LME boundaries can be found online at the addresses listed in Methods. Shoreline data used in Figs. 1 and 2 were obtained from the Global Self-Consistent Hierarchical High-Resolution Shoreline database version 2.2.0 available at https://www.ngdc.noaa.gov/mgg/shorelines/data/gshhg/oldversions/. Bathymetry data used in Figs. 1 and 2 were obtained from the NOAA ETOPO1 global relief model available at https://www.ngdc.noaa.gov/mgg/global/global.html. MODIS Aqua ocean chlorophyll data used to determine the TZCF location in Fig. 1 were obtained from the NASA Ocean Color Level-3 browser (https://oceancolor.gsfc.nasa.gov/l3/).
