# Supplementary material for: Migratory strategies of juvenile northern fur seals (Callorhinus ursinus): bridging the gap between pups and adults
Source: Sci Rep. 2019 Sep 26;9:13921. doi: 10.1038/s41598-019-50230-z (PMC6763446; doi:10.1038/s41598-019-50230-z)
Supplement: Supplementary file 1 — Migratory strategies of juvenile northern fur seals (Callorhinus ursinus): bridging the gap between pups and adults [file 41598_2019_50230_MOESM1_ESM.docx]

**Migratory strategies of juvenile northern fur seals (*Callorhinus ursinus*): bridging the gap between pups and adults**

Tonya Zeppelin^1*^, Noel Pelland^1^, Jeremy Sterling^1^, Brian Brost^1^, Sharon Melin^1^, Devin Johnson^1,^ Mary-Anne Lea^2^, Rolf Ream^1^

^1^Marine Mammal Laboratory, Alaska Fisheries Science Center, National Marine Fisheries Service, 7600 Sand Point Way N.E., Seattle, WA. 98115 USA

^2^Ecology and Biodiversity Centre, Institute for Marine and Antarctic Studies, College of Science and Engineering, University of Tasmania, 20 Castray Esplanade, Hobart, TAS 7000, Australia

[^*^Tonya.Zeppelin@noaa.gov](mailto:*Tonya.Zeppelin@noaa.gov)

**Supplementary Tables**

Supplementary Table S1. Mean (standard deviation) across trips of trip duration, net distance traveled and maximum distances from the start point for juvenile NFS pre-migratory trips of at least 1 d duration originating on the Pribilofs, Bogoslof and San Miguel islands.

|  | Overall | Pribilofs | Bogoslof | San Miguel |
| --- | --- | --- | --- | --- |
| Trips (n) | 92 | 39 | 43 | 10 |
| Duration (d) | 16.8 (14.6) | 24.6 (14.4) | 7.2 (6.8) | 28.2 (14.3) |
| Net distance traveled (km) | 851.8 (774.2) | 1361.4 (836.8) | 348.7 (317.0) | 1027.7 (516.5) |
| Max distance from start point (km) | 282.2 (239.0) | 461.2 (231.3) | 105.7 (89.2) | 342.6 (151.1) |

Supplementary Table S2. All generalized linear mixed-effects models tested for log pre-migratory trip duration (d). Fixed effects included: sex, site at which the trip originated (Pribilofs, Bogoslof or San Miguel), year, and days since 1 October on which each trip began (day). Columns include: model formula, number of estimated parameters (K), corrected AIC value (AICc), -2 times the fitted log-likelihood (LL), and difference in AICc from the best model (ΔAICc). For this response variable, models with ∆AICc < 2.24 were considered as competitor models, based on the number of observations and parameters in the top model.

| Model | K | AICc | -2*LL | ΔAICc |
| --- | --- | --- | --- | --- |
| **site + day** | **4** | 216.34 | 207.88 | **0** |
| **sex + site + day** | **5** | 217.04 | 206.35 | **0.70** |
| **site + sex*day** | **6** | 217.56 | 204.57 | **1.22** |
| **site*day** | **6** | 217.94 | 204.95 | **1.60** |
| sex + yr + site + day | 6 | 219.08 | 206.09 | 2.74 |
| site | 3 | 232.27 | 225.99 | 15.93 |
| sex + site | 4 | 234.40 | 225.94 | 18.06 |
| sex*site | 6 | 238.05 | 225.06 | 21.71 |
| day | 2 | 243.79 | 239.66 | 27.45 |
| sex*day | 4 | 247.80 | 239.34 | 31.46 |
| yr | 2 | 264.87 | 260.74 | 48.53 |
| [null] | 1 | 269.81 | 267.77 | 53.47 |
| sex | 2 | 271.29 | 267.16 | 54.96 |

Supplementary Table S3. Models tested for departure from the Bering Sea for juveniles from the eastern Pacific stock. Fixed effects included: sex, site of last departure from land (Pribilofs or Bogoslof), capture year, mass anomaly at capture (mass minus an average by sex), number of days at sea (days), and average north-south winds in the first 10 d at sea. Columns include: model formula, number of estimated parameters (K), corrected AIC value (AICc), -2 times the fitted log-likelihood (LL), and difference in AICc from the best model (ΔAICc). For this response variable, models with ∆AICc < 2.01 were considered as competitor models, based on the number of observations and parameters in the top model.

| Model | K | AICc | -2*LL | ΔAICc |
| --- | --- | --- | --- | --- |
| **site*sex + site:days_at_sea + sex:days_at_sea + site:sex:days_at_sea** | **7** | **577.30** | **563.27** | **0** |
| **early_vwind + site*sex + site:days_at_sea + sex:days_at_sea + site:sex:days_at_sea** | **8** | **579.14** | **563.1** | **1.84** |
| **site*sex + site*days_at_sea + sex*days_at_sea + site:sex:days_at_sea** | **8** | **579.21** | **563.17** | **1.91** |
| **yr + site*sex + site:days_at_sea + sex:days_at_sea + site:sex:days_at_sea** | **8** | **579.22** | **563.18** | **1.92** |
| site + sex*days_at_sea | 5 | 583.07 | 573.06 | 5.77 |
| site*days_at_sea + sex*days_at_sea | 6 | 584.22 | 572.20 | 6.92 |
| site + early_vwind + sex*days_at_sea | 6 | 584.34 | 572.32 | 7.04 |
| sex*days_at_sea | 4 | 587.83 | 579.82 | 10.54 |
| site + sex*date | 5 | 588.17 | 578.16 | 10.88 |
| site*date + sex*date | 6 | 588.90 | 576.87 | 11.60 |
| site + early_vwind + sex*date | 6 | 589.44 | 577.42 | 12.14 |
| sex*date | 4 | 591.54 | 583.53 | 14.24 |
| Site | 2 | 592.84 | 588.84 | 15.54 |
| site + sex | 3 | 593.09 | 587.08 | 15.79 |
| site + sex + early_vwind | 4 | 594.60 | 586.59 | 17.31 |
| site + days_at_sea | 3 | 594.78 | 588.78 | 17.48 |
| site + yr | 3 | 594.84 | 588.84 | 17.55 |
| site + sex + days_at_sea | 4 | 595.06 | 587.05 | 17.76 |
| site + sex + yr | 4 | 595.09 | 587.08 | 17.79 |
| site + sex + mass_anom + early_vwind | 5 | 596.50 | 586.48 | 19.20 |
| site + sex + days_at_sea + early_vwind | 5 | 596.57 | 586.55 | 19.27 |
| sex + site*days_at_sea | 5 | 596.58 | 586.56 | 19.28 |
| site + sex + yr + early_vwind | 5 | 596.61 | 586.59 | 19.31 |
| site + sex + yr + mass_anom | 5 | 597.03 | 587.01 | 19.73 |
| sex + early_vwind + site*days_at_sea | 6 | 597.94 | 585.91 | 20.64 |
| sex + yr | 3 | 598.13 | 592.12 | 20.83 |
| [null] | 1 | 598.35 | 596.35 | 21.06 |
| site + early_vwind + sex*mass_anom | 6 | 598.40 | 586.38 | 21.11 |
| site + sex + mass_anom*early_vwind | 6 | 598.45 | 586.43 | 21.15 |
| Yr | 2 | 598.47 | 594.47 | 21.17 |
| site + sex + days_at_sea + mass_anom + early_vwind | 6 | 598.48 | 586.46 | 21.18 |
| site + mass_anom + sex*early_vwind | 6 | 598.49 | 586.47 | 21.19 |
| site + sex + yr + mass_anom + early_vwind | 6 | 598.50 | 586.48 | 21.20 |
| days_at_sea | 2 | 599.72 | 595.72 | 22.42 |
| site + sex + yr + days_at_sea + mass_anom + early_vwind | 7 | 600.47 | 586.44 | 23.18 |

Supplementary Table S4. Models tested for average SST in the first 30 and 120 d of migration for juveniles from the eastern Pacific stock. Fixed effects included: sex, site of last departure from land (Pribilofs or Bogoslof), capture year, mass anomaly at capture (mass minus an average by sex), and day of departure since 1 October (day). Columns include: model formula, number of estimated parameters (K), corrected AIC value (AICc), -2 times the fitted log-likelihood (LL), and difference in AICc from the best model (ΔAICc). For T_30_, models with ∆AICc < 2.42 were considered as competitor models, based on the number of observations and parameters in the top model; for T_120_, the threshold was 2.49.

| Response variable | Model | K | AICc | -2*LL | ΔAICc |
| --- | --- | --- | --- | --- | --- |
| Ave SST (T_30_) | **sex + yr + day** | **4** | **141.86** | **133.06** | 0 |
|  | **sex + yr + site + day** | **5** | **144.27** | **133.05** | 2.41 |
|  | yr + site + day | 4 | 144.66 | 135.86 | 2.80 |
|  | mass + sex + yr + site + day | 6 | 146.23 | 132.48 | 4.37 |
|  | site + day + sex*yr | 6 | 146.29 | 132.54 | 4.43 |
|  | sex + day | 3 | 146.43 | 139.96 | 4.57 |
|  | yr + day + sex*site | 6 | 146.49 | 132.74 | 4.63 |
|  | day + mass*sex | 5 | 147.32 | 136.09 | 5.46 |
|  | day | 2 | 147.81 | 143.58 | 5.95 |
|  | mass + sex + day | 4 | 148.74 | 139.94 | 6.87 |
|  | mass + day | 3 | 149.62 | 143.15 | 7.76 |
|  | sex | 2 | 182.19 | 177.96 | 40.33 |
|  | mass + sex | 3 | 184.22 | 177.75 | 42.36 |
|  | sex + site | 3 | 184.27 | 177.80 | 42.41 |
|  | sex + yr | 3 | 184.41 | 177.94 | 42.55 |
|  | [null] | 1 | 185.81 | 183.74 | 43.95 |
|  | mass*sex | 4 | 186.32 | 177.52 | 44.46 |
|  | mass + sex + site | 4 | 186.34 | 177.54 | 44.48 |
|  | sex + yr + site | 4 | 186.44 | 177.64 | 44.57 |
|  | mass + sex + yr | 4 | 186.55 | 177.75 | 44.69 |
|  | sex*yr | 4 | 186.68 | 177.88 | 44.82 |
|  | mass | 2 | 186.73 | 182.50 | 44.87 |
|  | site | 2 | 187.53 | 183.29 | 45.66 |
|  | yr | 2 | 187.87 | 183.64 | 46.01 |
|  | yr + mass*sex | 5 | 188.75 | 177.52 | 46.89 |
|  | yr + site | 3 | 189.27 | 182.80 | 47.41 |
|  | yr + site + mass*sex | 6 | 190.95 | 177.20 | 49.09 |
| Ave SST (T_120_) | **sex + day** | **3** | **136.89** | 130.2 | 0 |
|  | **sex** | **2** | **136.92** | 132.59 | 0.04 |
|  | **mass + sex** | **3** | **137.86** | 131.17 | 0.97 |
|  | **mass + sex + day** | **4** | **138.16** | 128.98 | 1.27 |
|  | **sex + site** | **3** | **138.40** | 131.71 | 1.51 |
|  | **sex + yr + day** | **4** | **139.11** | 129.93 | 2.22 |
|  | **sex + yr** | **3** | **139.27** | 132.58 | 2.38 |
|  | mass + sex + site | 4 | 139.50 | 130.33 | 2.62 |
|  | mass + sex + yr | 4 | 140.24 | 131.06 | 3.35 |
|  | mass*sex | 4 | 140.35 | 131.17 | 3.46 |
|  | day + mass*sex | 5 | 140.55 | 128.73 | 3.67 |
|  | sex + yr + site | 4 | 140.68 | 131.50 | 3.79 |
|  | sex + yr + site + day | 5 | 140.98 | 129.17 | 4.10 |
|  | sex*yr | 4 | 141.71 | 132.54 | 4.83 |
|  | yr + day + sex*site | 6 | 141.84 | 127.21 | 4.95 |
|  | yr + mass*sex | 5 | 142.88 | 131.06 | 5.99 |
|  | mass + sex + yr + site + day | 6 | 142.96 | 128.34 | 6.08 |
|  | site + day + sex*yr | 6 | 143.78 | 129.16 | 6.89 |
|  | yr + site + mass*sex | 6 | 144.86 | 130.24 | 7.97 |
|  | mass | 2 | 152.37 | 148.03 | 15.48 |
|  | mass + day | 3 | 152.66 | 145.98 | 15.77 |
|  | day | 2 | 156.54 | 152.21 | 19.65 |
|  | [null] | 1 | 156.88 | 154.78 | 20.00 |
|  | site | 2 | 157.93 | 153.59 | 21.04 |
|  | yr + site + day | 4 | 158.43 | 149.25 | 21.54 |
|  | yr | 2 | 158.90 | 154.57 | 22.02 |
|  | yr + site | 3 | 158.93 | 152.24 | 22.04 |

Supplementary Table S5. Models of log mean dive depth (m) per 6-h dive bin for each individual during migration. Fixed effects included: lunar illumination fraction (lunar), proportion daylight (daylight), day of departure since 1 October (season) and large marine ecosystem (lme; Bering Sea Basin, Bering Sea Shelf, California Current, North Pacific and Gulf of Alaska). Columns include: model formula, number of estimated parameters (K), AIC value (AIC), -2 times the fitted log-likelihood (LL), and difference in AIC from the best model (ΔAIC).

| Model | K | AIC | -2*LL | ΔAIC |
| --- | --- | --- | --- | --- |
| **season + lme*lunar + lme*daylight + lunar*daylight** | **352** | **18599.94** | **17767.94** | **0** |
| season + lme*daylight + lunar*daylight | 288 | 18729.24 | 18025.24 | 129.30 |
| lme + season + lunar*daylight | 224 | 18739.82 | 18163.82 | 139.88 |
| lme*lunar + lme*daylight + lunar*daylight | 320 | 18826.84 | 18058.84 | 226.90 |
| lme*lunar + lunar*daylight | 256 | 18854.25 | 18214.26 | 254.32 |
| lme + season + lme*lunar + lme*daylight | 320 | 18880.73 | 18112.74 | 280.80 |
| season + lunar*daylight | 160 | 18891.87 | 18443.86 | 291.93 |
| lme*daylight + lunar*daylight | 256 | 18949.43 | 18309.42 | 349.49 |
| lme + lunar*daylight | 192 | 18965.07 | 18453.06 | 365.13 |
| lme + lunar + daylight + season | 192 | 19054.15 | 18542.14 | 454.21 |
| lme*lunar + lme*daylight | 288 | 19111.32 | 18407.32 | 511.38 |
| daylight + lme*lunar | 224 | 19170.04 | 18594.04 | 570.10 |
| lme + lunar + daylight | 160 | 19284.98 | 18836.98 | 685.05 |

Supplementary Table S6. Meta-analyses for synthesizing individual-level parameter estimates related to dive depth. All meta-analyses provide population-level inference (i.e., average effect across all individuals) concerning the parameters included in the AIC-best model for dive depth (i.e., LME, season, lunar fraction, proportion daylight, and the two-way interactions between LME, lunar fraction, and proportion daylight), and optionally include additional individual-level predictors as indicated by the ‘Model’ column. For example, inclusion of the covariate “stock” in the meta-analysis estimates an additional population-level effect describing the difference in dive behavior between individuals originating from the California versus eastern Pacific stocks. Similarly, the interaction “lme:sex” allows the population-level effect of LME to vary by sex. Other columns describe the number of parameters (K), AIC value (AIC), -2 times the fitted log-likelihood (LL), and difference in AIC from the best model (ΔAIC).

| Model | K | AIC | -2*LL | ΔAIC |
| --- | --- | --- | --- | --- |
| **sex + stock + year** | **173** | **3591.0** | **3245.0** | **0** |
| sex + stock | 172 | 3593.5 | 3249.6 | 2.5 |
| stock + year + lme:sex | 177 | 3594.0 | 3240.0 | 3.0 |
| stock + lme:sex | 176 | 3594.3 | 3242.4 | 3.3 |
| [null] | 170 | 3596.2 | 3256.2 | 5.2 |
| Sex | 171 | 3597.8 | 3255.8 | 6.8 |
| lme:sex | 175 | 3604.2 | 3254.2 | 13.2 |

Supplementary Table S7. Coefficients, standard errors and 95% confidence intervals describing the population-level effects of ecosystem (Bering Sea Basin, Bering Sea Shelf, California Current, Gulf of Alaska, North Pacific), season, lunar fraction, proportion daylight, sex, stock (California [San Miguel] or eastern Pacific stock) and year on juvenile northern fur seal dive depth (m) during migration.

| Coefficient | Estimate | se | 95% CI |
| --- | --- | --- | --- |
| Bering Sea Basin | 1.57 | 0.11 | (1.36, 1.77) |
| Bering Sea Shelf | 1.50 | 0.09 | (1.32, 1.69) |
| Califonia Current | 2.13 | 0.11 | (1.92, 2.34) |
| Gulf of Alaska | 2.14 | 0.16 | (1.83, 2.46) |
| North Pacific | 1.64 | 0.11 | (1.41, 1.86) |
| Season | 0.03 | 0.06 | (-0.09, 0.14) |
| Bering Sea Basin:lunar | 0.86 | 0.15 | (0.57, 1.15) |
| Bering Sea Shelf:lunar | 0.63 | 0.13 | (0.39, 0.88) |
| California Current:lunar | 0.74 | 0.09 | (0.55, 0.92) |
| Gulf of Alaska:lunar | 0.60 | 0.14 | (0.32, 0.88) |
| North Pacific:lunar | 0.74 | 0.10 | (0.55, 0.93) |
| Bering Sea Basin:daylight | 0.35 | 0.10 | (0.16, 0.54) |
| Bering Sea Shelf:daylight | 0.23 | 0.09 | (0.06, 0.41) |
| California Current:daylight | 0.15 | 0.13 | (-0.10, 0.39) |
| Gulf of Alaska:daylight | 0.08 | 0.10 | (-0.11, 0.27) |
| North Pacific:daylight | 0.17 | 0.07 | (0.04, 0.31) |
| lunar:daylight | -0.61 | 0.08 | (-0.77, -0.44) |
| Male | 0.19 | 0.06 | (0.07, 0.31) |
| San Miguel | -0.59 | 0.09 | (-0.75, -0.42) |
| year2007 | 0.25 | 0.05 | (0.15, 0.36) |

Supplementary Table S8. Models of log mean dive duration (s) per 6-h dive bin for each individual during migration. Fixed effects included: lunar illumination fraction (lunar), proportion daylight (daylight), day of departure since 1 October (season) and large marine ecosystem (lme). Columns include: model formula, number of estimated parameters (K), AIC value (AIC), -2 times the fitted log-likelihood (LL), and difference in AIC from the best model (ΔAIC).

| Model | K | AIC | -2*LL | ΔAIC |
| --- | --- | --- | --- | --- |
| **lme + season + lunar*daylight** | **223** | **12938.67** | **12364.66** | **0** |
| season + lme*lunar + lme*daylight + lunar*daylight | 349 | 12944.00 | 12117.98 | 5.32 |
| season + lunar*daylight | 160 | 12988.27 | 12540.28 | 49.61 |
| season + lme*daylight + lunar*daylight | 286 | 13013.11 | 12313.12 | 74.44 |
| lme*lunar + lunar*daylight | 254 | 13064.43 | 12428.42 | 125.76 |
| lme + lunar*daylight | 191 | 13125.73 | 12615.72 | 187.06 |
| lme*lunar + lme*daylight + lunar*daylight | 317 | 13127.58 | 12365.58 | 188.91 |
| lme*daylight + lunar*daylight | 254 | 13192.34 | 12556.34 | 253.67 |
| season + lme*lunar + lme*daylight | 317 | 13325.39 | 12563.40 | 386.72 |
| lme + lunar + daylight + season | 191 | 13372.91 | 12862.90 | 434.24 |
| daylight + lme*lunar | 222 | 13484.27 | 12912.26 | 545.60 |
| lme*lunar + lme*daylight | 285 | 13503.83 | 12805.82 | 565.16 |
| lme + lunar + daylight | 159 | 13553.63 | 13107.62 | 614.96 |

Supplementary Table S9. Meta-analyses for synthesizing individual-level parameter estimates related to dive duration. All meta-analyses provide population-level inference (i.e., average effect across all individuals) concerning the parameters included in the AIC-best model for dive depth (i.e., LME, season, lunar fraction, proportion daylight, and the two-way interactions between lunar fraction and proportion daylight), and optionally include additional individual-level predictors as indicated by the ‘Model’ column. For example, inclusion of the covariate “stock” in the meta-analysis estimates an additional population-level effect describing the difference in dive behavior between individuals originating from the California versus eastern Pacific stocks. Similarly, the interaction “lme:sex” allows the population-level effect of LME to vary by sex.Other columns describe the number of parameters (K), AIC value (AIC), -2 times the fitted log-likelihood (LL), and difference in AIC from the best model (ΔAIC).

| Model | K | AIC | -2*LL | ΔAIC |
| --- | --- | --- | --- | --- |
| **stock + lme:sex** | **60** | **1003.1** | **883** | **0** |
| sex | 55 | 1003.3 | 893.4 | 0.2 |
| sex + stock | 56 | 1003.4 | 891.4 | 0.3 |
| lme:sex | 59 | 1005.2 | 887.2 | 2.1 |
| stock + year + lme:sex | 61 | 1005.5 | 883.6 | 2.4 |
| [null] | 54 | 1005.6 | 897.6 | 2.5 |
| sex + stock + year | 57 | 1005.6 | 891.6 | 2.5 |

Supplementary Table S10. Coefficients, standard errors and 95% confidence intervals describing the population level effects of LME, season, lunar fraction, proportion daylight, sex and stock on juvenile northern fur seal dive duration (s) during migration.

| Coefficient | Estimate | se | 95% CI |
| --- | --- | --- | --- |
| Bering Sea Basin | 3.56 | 0.07 | (3.42, 3.71) |
| Bering Sea Shelf | 3.32 | 0.08 | (3.16, 3.48) |
| California Current | 3.72 | 0.09 | (3.54, 3.89) |
| Gulf of Alaska | 3.72 | 0.11 | (3.49, 3.94) |
| North Pacific | 3.47 | 0.07 | (3.33, 3.61) |
| Lunar | 0.73 | 0.05 | (0.62, 0.83) |
| Daylight | 0.45 | 0.05 | (0.35, 0.54) |
| Season | -0.01 | 0.04 | (-0.10, 0.07) |
| lunar:daylight | -0.63 | 0.06 | (-0.74, -0.52) |
| San Miguel | -0.18 | 0.05 | (-0.28, -0.07) |
| Bering Sea Basin:male | 0.10 | 0.07 | (-0.04, 0.23) |
| Bering Sea Shelf:male | 0.20 | 0.09 | (0.04, 0.37) |
| California Current:male | 0.41 | 0.16 | (0.09, 0.73) |
| Gulf of Alaska:male | 0.01 | 0.07 | (-0.12, 0.14) |
| North Pacific:male | 0.19 | 0.06 | (0.07, 0.31) |

Supplementary Table S11. Number, sex (female [F] or male [M]), instrument type (Kiwisat202 [Sirtrak, New Zealand], SPLASH or SPOT [Wildlife Computers, USA] satellite transmitters, year and breeding island of juvenile northern fur seal deployments.

| Year | Instrument type | St. Paul Island | Bogoslof  Island | San Miguel  Island | Total |
| --- | --- | --- | --- | --- | --- |
| 2006 | Kiwisat202 | 10F, 3M | 1F, 1M |  | 11F, 4M |
| 2006 | SPLASH | 4F, 4M | 4F, 4M |  | 8F, 8M |
| 2007 | SPOT | 11F, 9M |  |  | 11F, 9M |
| 2007 | SPLASH | 6F, 8M |  | 4F, 2M | 10F, 10M |


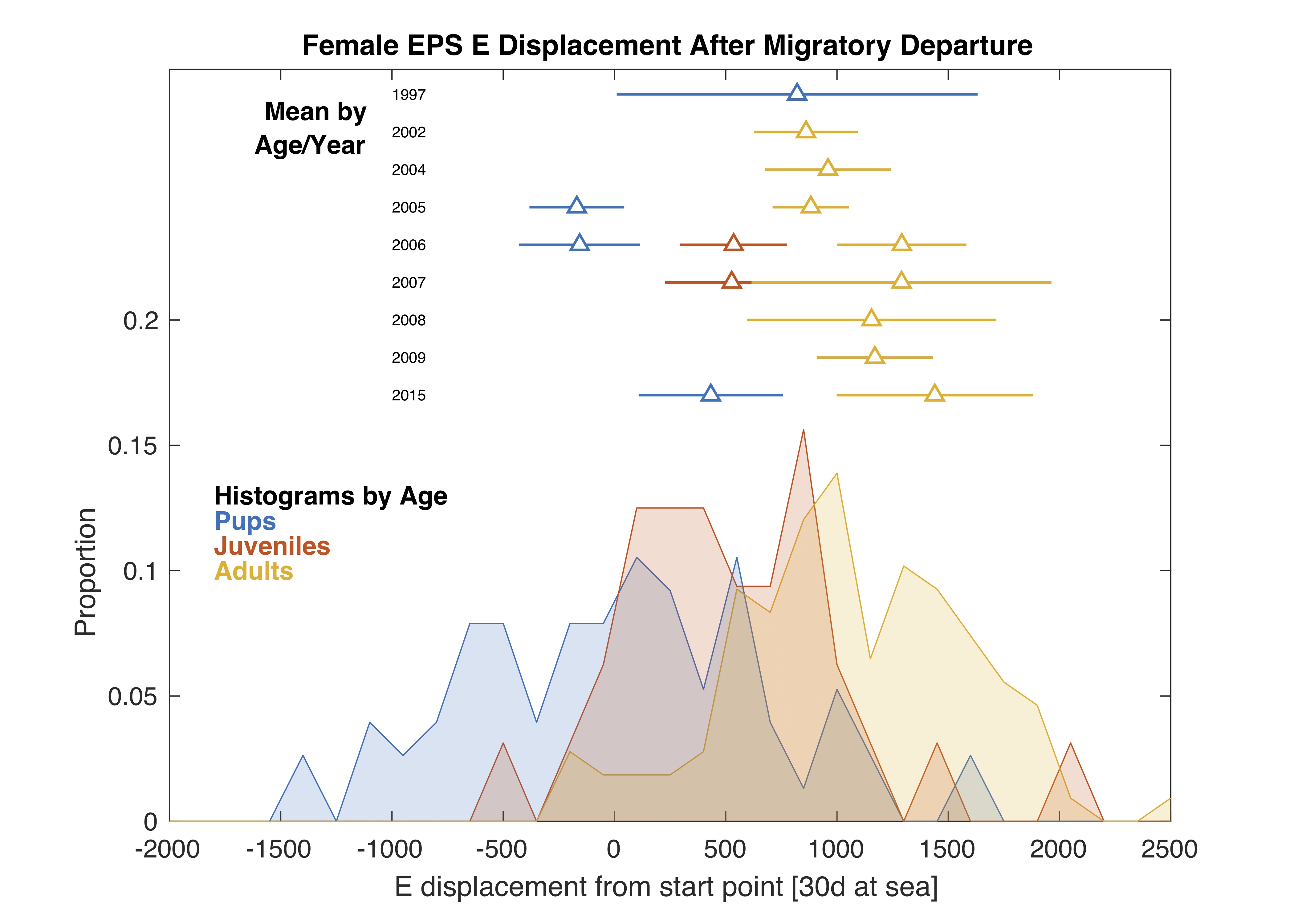


Supplementary Figure S1. Eastward displacement from departure site in the first 30 d at sea by age and year for migratory female northern fur seals of the eastern Pacific stock. Only tracks recording at least 30 d of migration are shown.  Shaded polygons at bottom show histograms of eastward displacement by age across all years, with proportion in 150 km bins indicated on the y-axis.  Triangles at top indicate average displacement by year for each age class, while whiskers indicate 95% confidence intervals.  Multiple age classes were sampled in 2005, 2006, 2007, and 2015.  The relationships between ages in eastward displacement during the first month at sea are consistently observed within years, including in 2006 when all three age classes were sampled.


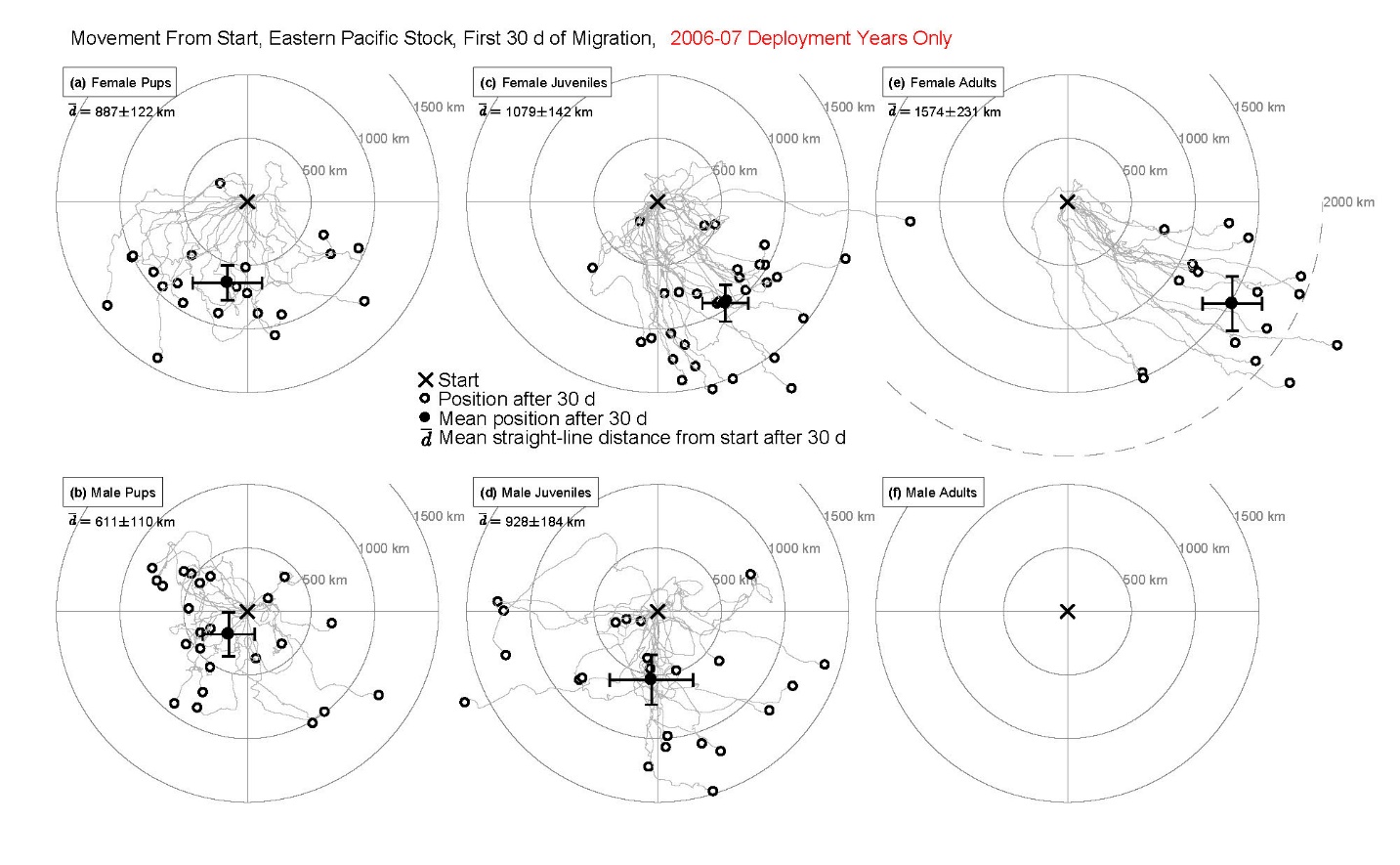


Supplementary Figure S2. Displacement from start point in the first 30 d at sea for migratory northern fur seals of the eastern Pacific stock for 2006-07 deployment years only. Only tracks recording at least 30 d of migration are shown. Left column (a-b) shows pups, central column (c-d) juveniles, and right column (e-f) adults; top row (a,c,e) is females, whereas bottom row (b,d) is males. No adult males were instrumented during these years. Within each group, the first at-sea point for each track is located at the origin ("x" marker), and displacements to the east (north) are along the positive x-(y-) axis. Filled white circles indicate the x/y position relative to the starting point for each animal at the end of 30 d; solid black circles indicate the average of these points for each group, with 95% confidence intervals (CI) indicated by whiskers. For each group, the average straight-line distance from the start point after 30 d and its CI is also shown.

Supplementary Figure S3. Composite proportional use of large marine ecosystems (LMEs) through time during the migration of eastern Pacific stock northern fur seals for 2006-07 deployments. Each of the first four panels corresponds to one LME or group of LMEs (Bering Sea Basin + Shelf [Bering], interior North Pacific Ocean [INP], Gulf of Alaska [GOA], California Current [CC]); lines within these plots indicate the proportion of tagged animals in each age/sex class observed within that LME versus day of year. Lower panel indicates number of tagged animals in each class versus day of year. Observed proportions have been smoothed with a 5 d half-width triangular-weight running average filter for clarity.
